# Testicular Hamartomas and Epididymal Tumor in a Cowden Disease: A Case Report

**DOI:** 10.1155/2010/135029

**Published:** 2010-06-09

**Authors:** Darshana D. Rasalkar, Bhawan K. Paunipagar

**Affiliations:** Departments of Diagnostic Radiology and Organ Imaging, Prince of Wales Hospital, The Chinese University of Hong Kong, Ngan Shing Street, Shatin, New Territories, Hong Kong

## Abstract

Testicular hamartomas (TH) is a benign condition. An association of TH with Cowden disease (CD) is known. Ultrasound features of hamartomas are often diagnostic. We present a case of Cowden disease with TH and an epididymal tumor. Imaging features of TH and its differentials has been discussed. Although, association of Cowden disease with many malignancies have been documented, epididymal tumor has not been described. To our knowledge, this paper is the first to describe epididymal tumor in association with Cowden disease.

## 1. Introduction

Cowden disease (CD) is a rare genetic condition associated with increased cellular proliferation of ectodermal, mesodermal, and endodermal tissues with incomplete penetrance, variable expressivity, and a gene mutation (PTEN-Phosphatase and Tensin Homologue) [1–4]. The prevalence rate of the Cowden disease is low with recent estimates at 1 per 250 000 [3].

Multiple hamartomas and malignant neoplasms are therefore known to occur with CD. Among malignancies cancer of the breast, thyroid, endometrium, and skin are more frequent. Space-occupying lesions in the liver and bone; abnormalities of the central nervous system have also been described [1]. 

In addition to the Cowden syndrome, Bannayan-Riley-Ruvalcaba syndrome, Proteus syndrome, and Proteus-like syndrome are grouped into PTEN hamartoma tumor syndrome (PHTS). Because of associated risk of malignancy in Cowden syndrome, follow up cancer surveillance based on their accompanying phenotypic features is recommended in individuals with PTEN hamartoma tumor syndrome [5].

## 2. Case Report

**Figure 1:**
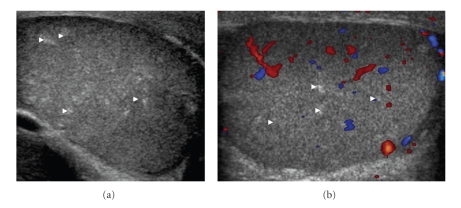
26-year-old patient presented with painless swelling in the right scrotum. (a) Grey Scale Ultrasound of Scrotum showing multiple small ill defined and randomly distributed echogenic foci within the testicular substances (white arrowheads). (b) Colour Doppler showing normal vascularity of testis. However, the lesions are typically avascular.

**Figure 2:**
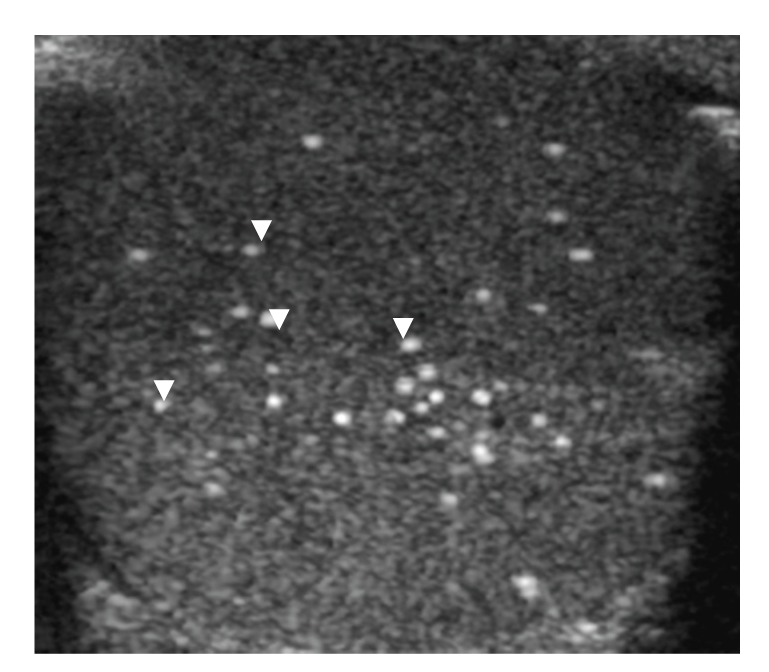
Grey scale transcrotal axial ultrasound of another patient with testicular microlithiasis. Note the multiple tiny densely echogenic, punctate and well-defined lesions (white arrowheads) easily distinguishable from those in testicular hamartomas in Figure 1.

**Figure 3:**
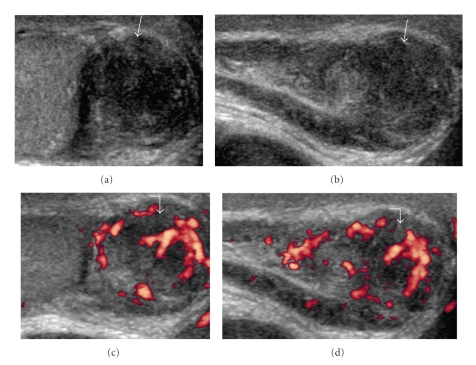
(a) (transverse), (b) (longitudinal) Grey-scale ultrasound images showing hypoechoic roundish mass (1.5 × 1.5 × 1.8 cm) in the tail of the right epididymis (white arrows). *Note*. The moderate vascularity on Power Doppler (c) (transverse), (d) (longitudinal) ultrasound (white curved arrows).

26-year-old gentleman with known Cowden disease, presented with recent onset of painless scrotal swelling. On examination, there was firm palpable mass at the right scrotum. Ultrasound showed multiple small ill-defined avascular echogenic foci in both testicular substances (Figures 1(a) and 1(b)), quite distinct from those seen in testicular microlithiasis (Figure 2). In addition, a hypoechoic lesion measuring (1.5 × 1.5 × 1.8 cm) was identified in the tail of the right epididymis (Figures 3(a) and 3(b)). Moderate vascularity was demonstrable on power Doppler (Figures 3(c) and 3(d)). Based on history and sonological findings, diagnosis of testicular hamartomas was certain. The size and vascularity of the right epididymal lesion pointed to underlying epididymal tumor. Further biopsy also confirmed the diagnosis of an adenomatoid tumor of the right epididymis.

## 3. Discussion

The diagnosis of CS has been facilitated by the establishment of the International Cowden Consortium, which proposed a set of operational diagnosis criteria divided into pathognomonic lesions (facial trichilemmomas, acral keratosis, papillomatous papules, and mucous lesions), major criteria (mammary carcinoma, thyroid carcinoma, macrocephalia, Lhermitte-Duclos disease, and endometrial carcinoma) and minor criteria (other thyroid pathology, mental retardation, intestinal hamartomatous polyps, fibrocystic mammary disease, lipomas, fibromas, urogenital tumors, and urogenital malformations) [6].

Cowden syndrome is diagnosed when the patient shows the presence of (1) pathognomonic lesions, (six or more facial papules, of which three or more must be trichilemmomas; facial papules and papillomatosis in oral mucosa; papillomatosis in oral mucosa and acral keratosis; six or more palmoplantar keratosis lesions); (2) presence of two major criteria, one of which must be macrocephalia or Lhermitte-Duclos disease; (3) presence of one major criterion and three minor criteria; (4) presence of four minor criteria [6].

Lindsay et al. (2003) [7] first described multiple bilateral testicular lesions in CD, the lesions were assumed to be hamartomas based on having fat component on MRI, although no histopathology evidence was documented. Thereafter, in 2006, Woodhouse et al. [8] published a large cohort of 8 CD patients, all having multiple testicular lesions with histological proof of lipomatous hamartomas in 4 patients. The typical imaging features were described as multiple avascular hyperechogenic foci of approximately 1–6 mm size. Most lesions are small, nonshadowing and randomly distributed within both testicles [8]. Similar imaging features were seen in our case.

Microlithiasis, is the most commonly documented testicular pathology occurring as a multiple randomly scattered punctate lesions in the testicular parenchyma. It is characterized by several tiny (1 to 3 mm) echogenic foci with variable acoustic shadowing. These represent calcified deposits in the lumen of seminiferous tubules and therefore have a very high echogenicity distinct to the lesions in the Cowden's testes (Figure 2). Lymphoma, leukaemia, and metastatic disease can occur as a focal or multiple hypoechoic lesion to a diffusely altered echogenicity [9, 10] often can be distinguishable from the testicular lipomatosis.

Epididydimal tumors are a rare with the adenomatoid tumor being most common. Two other benign tumors of the epididymis are leiomyomas and papillary cystadenomas [11]. 

The tumor has three basic patterns: tubules, cords, and small nests, formed of cells that are cuboidal with vacuolated cytoplasm. Peripheral eosinophilic and lymphatic infiltration is frequently noted [12]. Gaping spaces with necrotic tubular component and smaller spaces, representing ghost remnants of the typical vacuolar spaces, are major clues to the diagnosis. Immunohistochemical confirmation with mesothelial-related markers (calretinin, HMBE1) is helpful in the differential with nonmehothelial lesions including metastatic carcinoma, malignant mesothelioma, histiocytoid hemangioma, and carcinoma of the rete testis.

In addition, gray scale sonographic, color Doppler sonographic, and some clinical features may be helpful for differential diagnosis of focal epididymal lesions [13]. 

The tumors are smooth, round, and wellcircumscribed and can vary in size from a few millimeters up to 5 cm [14, 15]. Alleman et al. [13] have described the ultrasound features helpful in differential diagnosis of focal epididymal lesions. According to this study, most solid epididymal masses (94%) were benign. A size of greater than 1.5 cm and the presence of color Doppler flow may help identify possible malignant masses. Both of these features were present in our case (Figure 3), however biopsy of this mass confirmed the diagnosis of an adenomatoid tumor, a benign tumor of the epididymis. 

To conclude, the previous literature-based imaging features had helped us in the confident diagnosis of testicular hamartomas in Cowden disease. Although not documented, an epididymal tumor can be explained in this inheritable condition which itself is characterized by multiple associated hamartomatous and variable tumors elsewhere.
